# Phospholipid biomarkers of coronary heart disease

**DOI:** 10.1186/s40780-024-00344-y

**Published:** 2024-05-11

**Authors:** Shin-ya Morita

**Affiliations:** Department of Pharmacotherapeutics, Shiga University of Medical Science, Otsu, Shiga 520-2192 Japan

**Keywords:** Phospholipid, Cholesterol, Triglyceride, Lipoprotein, Coronary heart disease, Atherosclerosis, Enzymatic fluorometric assay, Phosphatidylcholine, Phosphatidylethanolamine, Sphingomyelin

## Abstract

Coronary heart disease, also known as ischemic heart disease, is induced by atherosclerosis, which is initiated by subendothelial retention of lipoproteins. Plasma lipoproteins, including high density lipoprotein, low density lipoprotein (LDL), very low density lipoprotein, and chylomicron, are composed of a surface monolayer containing phospholipids and cholesterol and a hydrophobic core containing triglycerides and cholesteryl esters. Phospholipids play a crucial role in the binding of apolipoproteins and enzymes to lipoprotein surfaces, thereby regulating lipoprotein metabolism. High LDL-cholesterol is a well-known risk factor for coronary heart disease, and statins reduce the risk of coronary heart disease by lowering LDL-cholesterol levels. In contrast, the relationships of phospholipids in plasma lipoproteins with coronary heart disease have not yet been established. To further clarify the physiological and pathological roles of phospholipids, we have developed the simple high-throughput assays for quantifying all major phospholipid classes, namely phosphatidylcholine, phosphatidylethanolamine, phosphatidylserine, phosphatidic acid, phosphatidylinositol, phosphatidylglycerol + cardiolipin, and sphingomyelin, using combinations of specific enzymes and a fluorogenic probe. These enzymatic fluorometric assays will be helpful in elucidating the associations between phospholipid classes in plasma lipoproteins and coronary heart disease and in identifying phospholipid biomarkers. This review describes recent progress in the identification of phospholipid biomarkers of coronary heart disease.

## Background

According to the World Health Organization, the most leading cause of deaths worldwide in 2019 was ischemic heart disease (16%), also called coronary heart disease. In Japan, heart disease was the second leading cause (14.8%) of death after cancers (24.6%) in 2022, and the prevalence of heart disease is currently increasing. Coronary heart disease, including angina pectoris and myocardial infarction, is caused by the atherosclerosis of the coronary arteries. The development of atherosclerosis involves many pathogenic processes, including lipoprotein subendothelial retention, modification and aggregation, macrophage chemotaxis, and foam cell formation [1]. Atherosclerosis is induced by the subendothelial retention of atherogenic lipoproteins containing apolipoprotein (apo) B, such as low density lipoproteins (LDL), lipoprotein remnants, and lipoprotein (a) (Lp(a)) [1, 2]. In the subendothelium, apoB-containing lipoprotein particles are modified by phospholipid (PL) hydrolysis, oxidation, proteolysis, glycosylation, aggregation, or complexation with proteoglycans [1]. Macrophage uptake of modified lipoproteins, but not native LDL, induces the formation of foam cells that accumulate cholesteryl esters (CEs). In contrast, high density lipoprotein (HDL) particles have atheroprotective functions and mediate the efflux of excess cholesterol from foam cells in atherosclerotic lesions [3].

### Lipoproteins and coronary heart disease

**Fig. 1:**
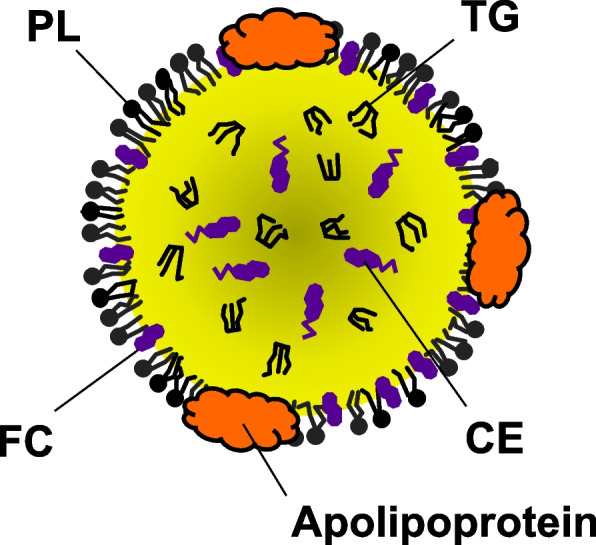
Model of plasma lipoprotein structure. A hydrophobic core consisting of TGs and CEs is surrounded by a surface monolayer composed of PLs, FC, and apolipoproteins. CE, cholesteryl ester; FC, free cholesterol; PL, phospholipid; TG, triglyceride

**Fig. 2:**
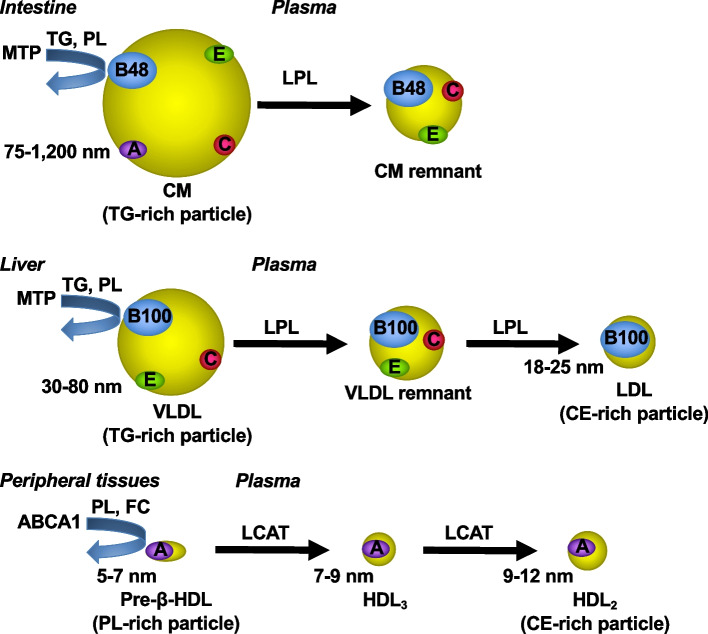
Metabolism of plasma lipoproteins. CM (diameter 75–1,200 nm) and VLDL (diameter 30–80 nm) are formed in the intestine and liver, respectively, in which TG and PL are carried to apoB by MTP. In the plasma, lipolysis by LPL mediates the conversion of CMs to CM remnants and that of VLDLs to VLDL remnants and subsequently to LDLs (diameter 18–25 nm). CM and VLDL particles are rich in TGs, and LDL particles are rich in CEs. CM remnants, VLDL remnants, and LDLs are removed from circulation by the liver. In the formation of HDL in peripheral tissues, the cellular efflux of PLs and FC is mediated by apoA-I and ABCA1. Through esterification of FC by LCAT, pre-β-HDL (diameter 5–7 nm) is converted to HDL_3_ (diameter 7–9 nm) and HDL_2_ (diameter 9–12 nm). CE, cholesteryl ester; CM, chylomicron; FC, free cholesterol; HDL, high density lipoprotein; LCAT, lecithin:cholesterol acyltransferase; LDL, low density lipoprotein; LPL, lipoprotein lipase; MTP, microsomal triglyceride transfer protein; PL, phospholipid; TG, triglyceride; VLDL, very low density lipoprotein

In the plasma lipoprotein structures, a hydrophobic core composed of triglycerides (TGs), also called triacylglycerols, and CEs is surrounded by a surface monolayer composed of PLs, unesterified (free) cholesterol (FC), and apolipoproteins (Fig. 1) [1]. Plasma lipoproteins are classified into HDL (diameter 5–12 nm, density 1.063–1.210 g/mL), LDL (diameter 18–25 nm, density 1.006–1.063 g/mL), very low density lipoprotein (VLDL) (diameter 30–80 nm, density 0.94–1.006 g/mL), and chylomicron (CM) (diameter 75–1,200 nm, density < 0.94 g/mL) (Fig. 2) [3]. Each LDL particle or VLDL particle contains one molecule of apoB100, and each CM particle contains a single molecule of apoB48. In contrast, HDL particles primarily contain apoA-I but not apoB. CM particles also contain ApoA-I. Lp(a) (diameter ~ 25 nm, density 1.05–1.1 g/mL) is an LDL-like particle containing apo(a) covalently bound to apoB-100 [4].

CM and VLDL particles are assembled in enterocytes and hepatocytes, respectively, where TG and PL molecules are transferred to apoB by microsomal TG transfer protein (MTP) (Fig. 2) [1]. The PL molecules in the CM and VLDL particles are derived from intracellular membranes, particularly the endoplasmic reticulum membrane. In the circulation, TG hydrolysis, also called lipolysis, by lipoprotein lipase (LPL) is activated by apoC-II on lipoprotein particles and mediates the conversion of CMs to CM remnants and that of VLDLs to VLDL remnants and subsequently to LDLs. The CM and VLDL particles are TG rich, whereas LDL is rich in CEs. The PL molecules in lipoprotein particles are hydrolyzed by hepatic TG lipase and LPL [5]. ApoC-II is responsible for the activation of LPL [6]. LDL particles and lipoprotein remnants are removed from circulation by the liver. ApoE on lipoprotein remnants enhances the binding to heparan sulfate proteoglycans on the hepatocyte surface and their uptake into the hepatocytes through LDL receptor or LDL receptor-related protein [1, 7, 8]. LDL receptor interacts with apoB-100 and apoE, but not apoB-48. Proteins on LDL particles are almost exclusively apoB-100, which promotes their internalization into the hepatocytes via LDL receptor [1]. Proprotein convertase subtilisin-kexin type 9 (PCSK9) binds to LDL receptor and prevents the recycling of internalized LDL receptor to the cell surface, leading to the intracellular degradation of LDL receptor [9]. ApoC-III inhibits lipolysis mediated by LPL and hepatic uptake of lipoproteins mediated by apoE or apoB-100 [1, 6–8]. The binding of apolipoproteins and enzymes to the lipoprotein surface is regulated by the PL composition of the surface monolayer, which determines lipoprotein metabolism.

During the formation of HDL particles containing apoA-I, the efflux of PLs and FC from the cell surface plasma membrane is mediated by the interaction of apoA-I with the ATP-binding cassette transporter ABCA1, which is the initial step of the reverse cholesterol transport pathway (Fig. 2) [10, 11]. The nascent form of HDL, pre-β-HDL (diameter 5–7 nm, density > 1.210 g/mL), includes discoidal particles containing two apoA-I molecules [3, 12]. By the action of lecithin:cholesterol acyltransferase (LCAT), pre-β-HDL particles are converted to spherical α-HDL particles, HDL_3_ (diameter 7–9 nm, density 1.125–1.210 g/mL) and HDL_2_ (diameter 9–12 nm, density 1.063–1.125 g/mL), and most of plasma HDL are spherical α-HDL particles [12]. On HDL particles, LCAT catalyzes the formation of CEs by the transesterification of a fatty acid from the *sn*-2 position of PC to the 3-hydroxyl group of FC [12]. The transformation of smaller HDL_3_ particles to larger HDL_2_ particles is promoted by the esterification of FC by LCAT. ApoA-I serves as the main activator of LCAT on the HDL surface [3, 12]. Cholesteryl ester transfer protein (CETP) facilitates the transfer of CEs from HDL to apoB-containing lipoproteins and that of TGs from apoB-containing lipoproteins to HDL [12]. Phospholipid transfer protein mediates the transfer of PLs from apoB-containing lipoproteins to HDL and that between HDL particles [3]. CEs and FC in HDL particles are transferred to hepatocytes through scavenger receptor B1-dependent selective uptake but not through whole particle uptake [3, 12].

The incidence of coronary heart disease is positively correlated with LDL-cholesterol levels, but negatively correlated with HDL-cholesterol levels [13]. The levels of LDL-cholesterol and HDL-cholesterol represents the sum of the concentrations of FC and CEs in LDL and HDL, respectively. Familial hypercholesterolemia, caused by a mutation in LDL receptor, is characterized by the elevated levels of LDL-cholesterol [1]. Tangier disease is characterized by a complete deficiency of ABCA1 function and extremely low levels of HDL [3, 10]. 3-Hydroxy-3-methylglutaryl-coenzyme A reductase inhibitors, such as pravastatin, simvastatin, fluvastatin, atorvastatin, pitavastatin, and rosuvastatin, lower LDL-cholesterol levels and reduce the risk of coronary heart disease [14–20]. Evolocumab, a monoclonal antibody against PCSK9, lowers the levels of LDL-cholesterol and Lp(a), and prevents cardiovascular events [21, 22]. In addition, the small interfering RNA inclisiran inhibits the hepatic synthesis of PCSK9 and reduces the level of LDL-cholesterol [23]. In patients with homozygous familial hypercholesterolemia, the MTP inhibitor lomitapide lowers LDL-cholesterol, VLDL-cholesterol, and plasma TG levels [24, 25]. The CETP inhibitor anacetrapib markedly increases HDL-cholesterol levels, but only slightly reduces coronary events [26]. The high level of plasma TG is a weak risk factor for coronary heart disease compared to the high level of LDL-cholesterol or the low level of HDL-cholesterol [27]. In fact, a potent selective peroxisome proliferator-activated receptor α modulator, pemafibrate, is ineffective in reducing the incidence of cardiovascular events despite the marked reduction in TG levels [28, 29]. In contrast, the relationships of PLs in plasma lipoproteins with coronary heart disease are not well understood.

### Phospholipid molecular structures

**Fig. 3:**
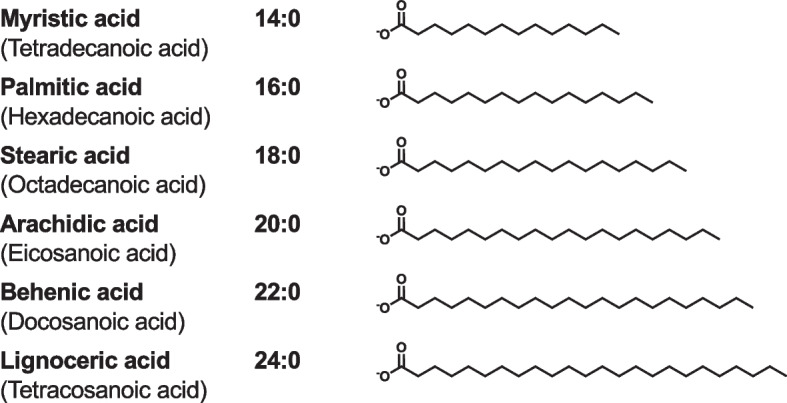
Chemical structures of saturated fatty acids. For example, 14:0 denotes 14-carbon chain with no double bond

**Fig. 4:**
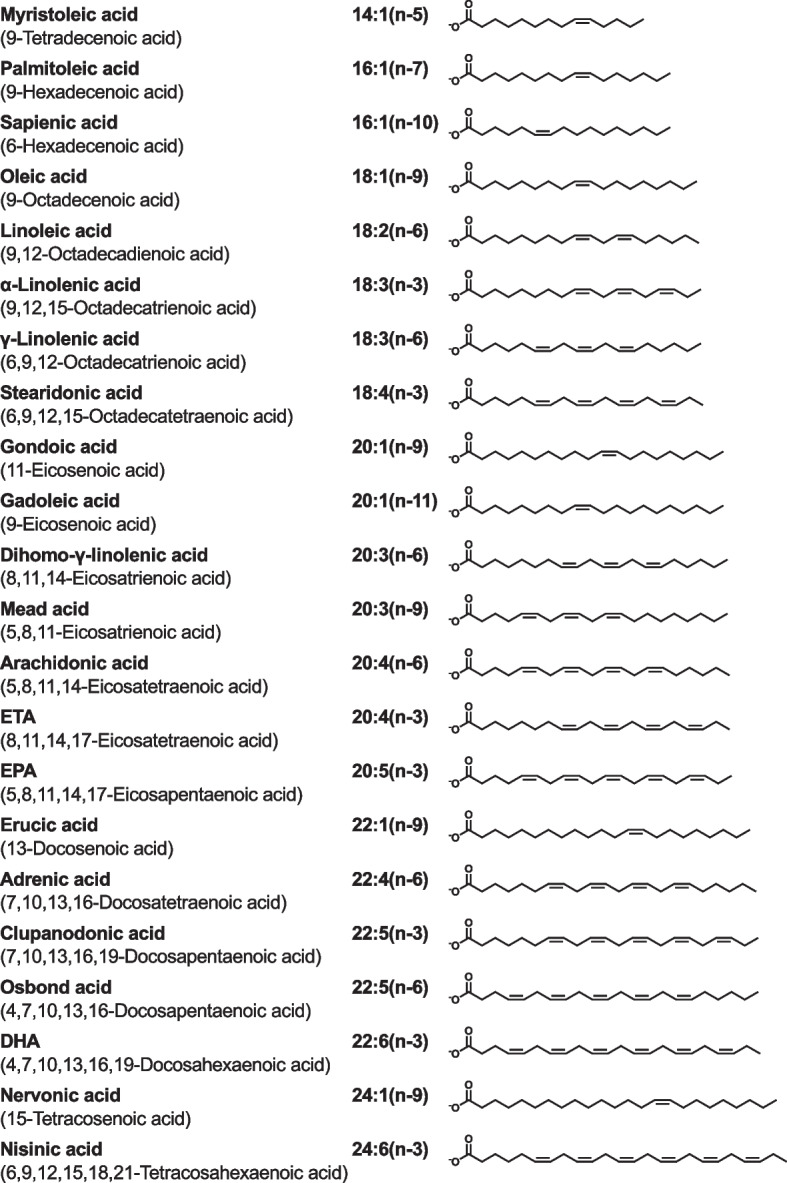
Chemical structures of unsaturated fatty acids. For example, 20:5(n-3) denotes 20-carbon chain with 5 double bonds and the first double bond in the 3rd position from the methyl end. DHA, docosahexaenoic acid; EPA, eicosapentaenoic acid; ETA, eicosatetraenoic acid

PL, consisting of two hydrophobic acyl chains and a hydrophilic head group, is an amphiphilic molecule comprising surface monolayers of plasma lipoprotein particles. In mammals, there are two groups of PLs: glycerophospholipids (GPLs) containing a glycerol backbone and sphingophospholipids (SPLs) containing a sphingosine backbone [30]. Various long-chain saturated, monounsaturated, and polyunsaturated fatty acids are esterified into PL molecules (Figs. 3 and 4). The chain length of fatty acids linked to PLs varies from 14 to 24 carbon atoms. In addition to chain length, fatty acids are classified according to the number, position, and stereochemistry (*cis* or *trans*) of their double bonds. Most of the double bonds in fatty acids are *cis*. For example, linoleic acid, also called 9,12-octadecadienoic acid, is denoted as 18:2(n-6), which means 18-carbon chain with 2 double bonds and the first double bond in the 6th position from the methyl end.

**Fig. 5:**
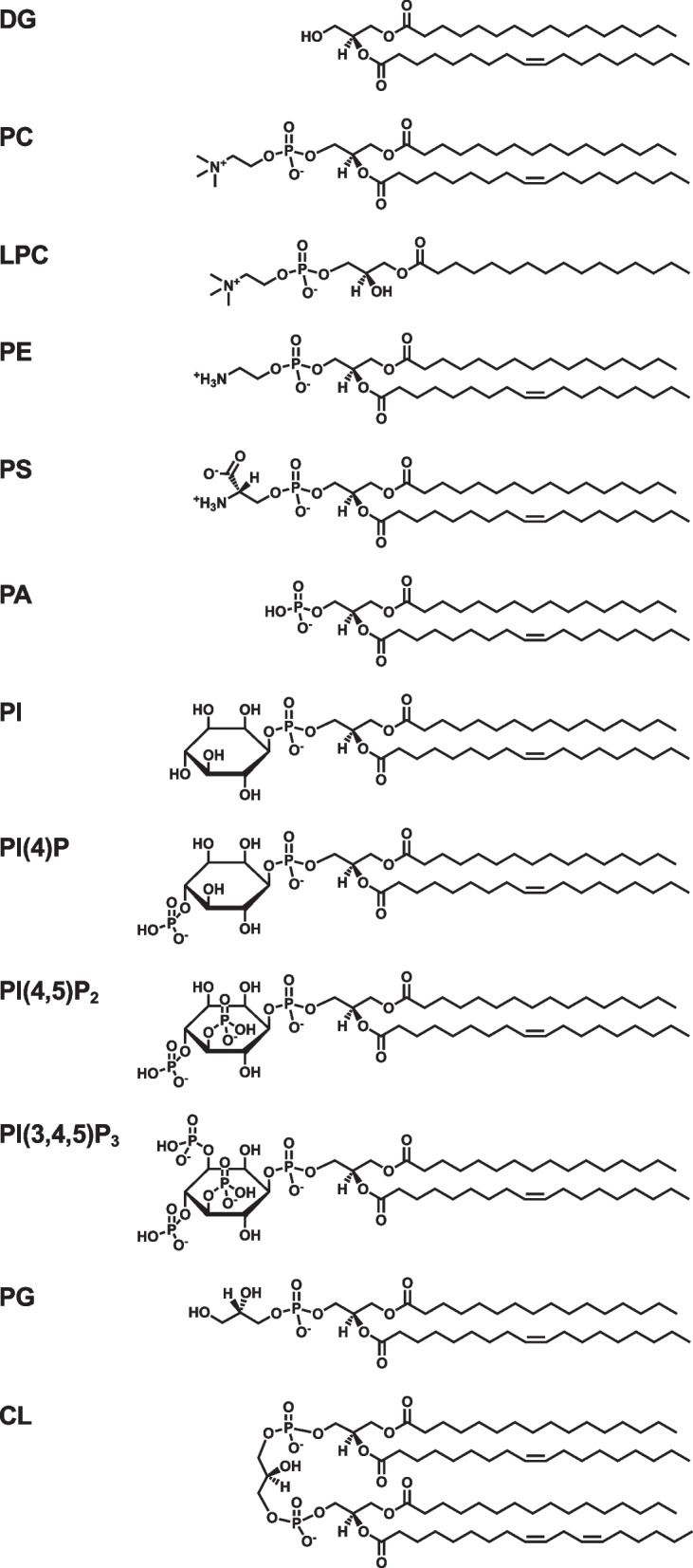
Chemical structures of glycerophospholipids and DG. Glycerophospholipids consist of a glycerol backbone, a hydrophilic head group, and two hydrophobic acyl chains. Based on the head group structures, glycerophospholipids are divided into classes, including PC, PE, PS, PA, PI, PG, and CL. Lysoglycerophospholipids, such as LPC, possess only one acyl chain. PI is further phosphorylated to form PI phosphates, including PI(4)P, PI(4,5)P_2_, and PI(3,4,5)P_3_. CL, cardiolipin; DG, diglyceride; LPC, lysophosphatidylcholine; PA, phosphatidic acid; PC, phosphatidylcholine; PE, phosphatidylethanolamine; PG, phosphatidylglycerol; PI, phosphatidylinositol; PS, phosphatidylserine

**Fig. 6:**
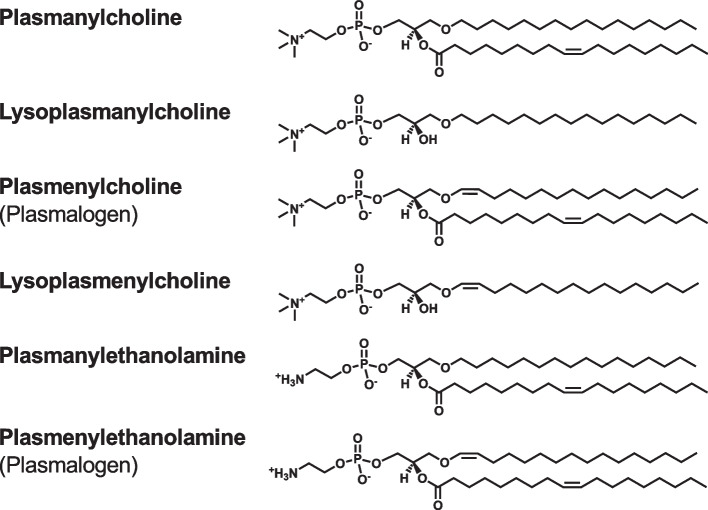
Chemical structures of ether-linked glycerophospholipids. Plasmanylcholine and plasmanylethanolamine contain an ether bond at the *sn*-1 position of the glycerol backbone. Plasmenylcholine and plasmenylethanolamine, also called plasmalogens, contain a vinyl-ether bond at the *sn*-1 position. Lysoplasmanylcholine and lysoplasmenylcholine possess one alkyl chain and one alkenyl chain, respectively

In the GPL structures, long-chain fatty acids are esterified to the *sn*-1 and *sn*-2 positions of the glycerol backbone. Based on the structures of the polar head groups linked via a phosphodiester bond to the *sn*-3 position of the glycerol backbone, GPLs are divided into different classes, such as phosphatidylcholine (PC), phosphatidylethanolamine (PE), phosphatidylserine (PS), phosphatidic acid (PA), phosphatidylinositol (PI), and phosphatidylglycerol (PG) (Fig. 5). In the structure of PA, only a small phosphate group is attached to the *sn*-3 position of the glycerol backbone of diglyceride (DG), also called diacylglycerol. The polar head groups of PC, PE, PS, PI, and PG are phosphocholine, phosphoethanolamine, phosphoserine, phosphoinositol, and phosphoglycerol, respectively. PI is further phosphorylated at the 3-, 4-, and 5-positions of the inositol ring to form PI phosphates, namely PI(3)P, PI(4)P, PI(5)P, PI(3,4)P_2_, PI(3,5)P_2_, PI(4,5)P_2_, and PI(3,4,5)P_3_. In the structure of cardiolipin (CL), two PA molecules are linked to one glycerol molecule. PS, PA, PI, PG, and CL are negatively charged GPLs. Lysoglycerophospholipids, including lysophosphatidylcholine (LPC), lysophosphatidylethanolamine (LPE), lysophosphatidylserine (LPS), lysophosphatidic acid (LPA), lysophosphatidylinositol (LPI), and lysophosphatidylglycerol (LPG), possess only one acyl chain. Plasmanylcholine and plasmanylethanolamine are ether-linked GPLs characterized by an ether bond at the *sn*-1 position of the glycerol backbone (Fig. 6). Plasmenylcholine and plasmenylethanolamine, also called plasmalogens, contain a vinyl-ether bond at the *sn*-1 position. Lysoplasmanylcholine and lysoplasmenylcholine have one alkyl chain and one alkenyl chain, respectively.

**Fig. 7:**
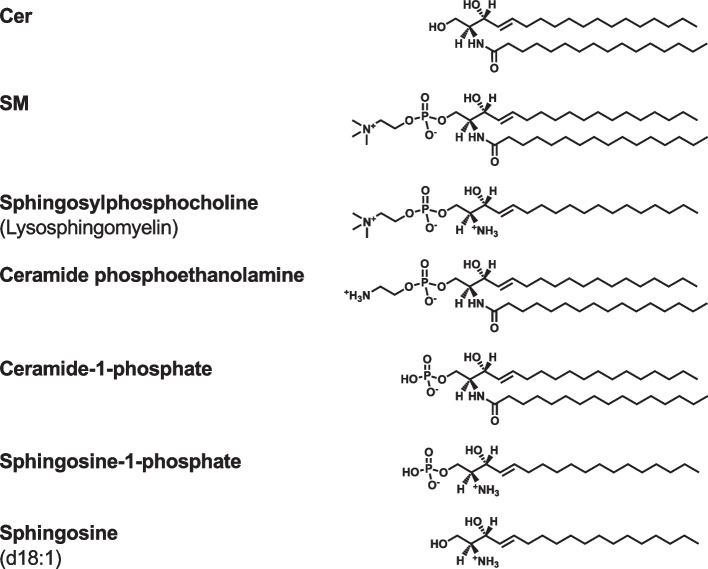
Chemical structures of sphingophospholipids, Cer, and sphingosine. Cer, SM, ceramide phosphoethanolamine, and ceramide-1-phosphate contain one fatty acid attached via an amide bond to the sphingosine backbone. Sphingosylphosphocholine and sphingosine-1-phosphate are lysosphingophospholipids. d18:1 denotes sphingosine containing 18 carbon atoms. Cer, ceramide; SM, sphingomyelin

In the molecular structures of SPLs, such as sphingomyelin (SM), ceramide phosphoethanolamine, and ceramide-1-phosphate, one fatty acid is attached via an amide bond to the sphingosine backbone (Fig. 7). In the structure of SM, the phosphocholine headgroup is bound to ceramide (Cer). Sphingosylphosphocholine and sphingosine-1-phosphate are lysosphingophospholipids. Sphingosine containing 18 carbon atoms (d18:1) is the most common sphingoid base.

Thus, a very wide variety of PL molecular species are present in the human body. The biosynthetic pathways of PLs have been summarized in our previous review [30].

### Phospholipids and coronary heart disease

Among PL classes in plasma lipoprotein particles, PC is the most abundant, and SM is the second most abundant [1, 3]. Other PL classes are quantitatively minor in the lipoprotein particles. The SM/PC ratio in LDL is higher than that in VLDL [1, 31, 32]. Jiang et al. have shown that the levels of plasma SM and the ratios of SM/(PC + SM) are positively associated with coronary heart disease, suggesting that SM is a risk factor [33].

Fernandez et al. have reported that SM (38:2) is associated with an increased risk of cardiovascular disease, whereas LPC (16:0 and 20:4) is associated with a decreased risk of cardiovascular disease [34]. The plasma level of PE (36:5) has been reported to be associated with cardiovascular diseases [35]. There are negative associations of plasma SM (28:1) and LPC (18:1 and 18:2) with incident coronary heart disease events [36]. Sigruener et al. have reported that coronary heart disease mortality is positively associated with the plasma concentrations of PC (30:1, 32:0, 34:1, 36:1, and 38:0), ether-linked PC (PC-O) (32:0, 32:1, 34:0, 34:1, and 38:5), PE (30:1, 32:1, 34:1, 34:2, 34:3, 36:1, 36:2, 36:3, 36:4, 38:2, and 40:1), ether-linked PE (PE-O) (32:0 and 36:2), SM (d18:1/16:0, d18:1/16:1, d18:1/24:1, and d18:1/24:2), and Cer (d18:1/16:0, d18:1/18:0, and d18:1/24:1), but inversely associated with those of PC (32:2, 36:4, 36:5, 38:3, 38:4, 38:5, 38:6, 38:7, 40:6, and 40:7), LPC (16:0 and 18:0), PE (36:6), PE-O (38:7), SM (d18:1/23:0, d18:1/23:1, and d18:1/24:0), and Cer (d18:1/23:0 and d18:1/24:0), suggesting that LPC species and highly polyunsaturated PC species have protective effects [37]. In patients with coronary heart disease, the concentration of Cer (d18:1/16:0) is associated with the occurrence of major adverse cardiac events [38, 39]. Havulinna et al. have reported that serum concentrations of Cer (d18:1/16:0, d18:1/18:0, and d18:1/24:1) are associated with the risk of incident major adverse coronary events in apparently healthy individuals [40]. In addition, Laaksonen et al. have shown that the ratios of Cer (d18:1/16:0)/Cer (d18:1/24:0), Cer (d18:1/18:0)/Cer (d18:1/24:0), and Cer (d18:1/24:1)/Cer (d18:1/24:0) in the plasma are risk predictors of cardiovascular death in patients with stable coronary heart disease and acute coronary syndromes [41]. Alshehry et al. have reported that, in patients with type 2 diabetes mellitus, the plasma levels of Cer (d18:1/24:1), SM (34:1), plasmanylcholine (32:0, 32:1, 34:1, 36:1, and 36:2), plasmenylcholine (32:1 and 34:1), LPC (20:1), and lysoplasmanylcholine (18:0, 18:1, 22:0, 22:1, 24:0, 24:1, and 24:2) were associated with future cardiovascular events, whereas the plasma levels of polyunsaturated acyl chain-containing PC (34:5, 35:4, and 40:6) and plasmenylcholine (36:5 and 38:6) were inversely associated with events [42].

For primary prevention, pravastatin reduces the risk of cardiovascular events. Changes in plasma PI (36:2) and PC (38:4) have been shown to be negatively and positively associated with pravastatin treatment, respectively, and positively and negatively associated with future cardiovascular events, respectively, independent of changes in LDL-cholesterol [43]. In contrast, the plasma levels of PI species are consistently lower in patients with coronary heart disease than in healthy individuals [44]. Idiopathic heart failure is largely attributable to coronary heart disease, pressure overload, and type 2 diabetes mellitus. The plasma concentrations of Cer (d18:1/16:0) and PC (32:0) have been shown to be associated with heart failure risk [45]. Jensen et al. have reported that the plasma concentrations of very-long chain saturated fatty acid (20:0, 22:0, or 24:0)-containing Cer and SM are associated with a reduced risk of atrial fibrillation, whereas those of palmitic acid (16:0)-containing Cer and SM are associated with an increased risk of atrial fibrillation [46]. Long-term high blood pressure is a risk factor for coronary heart disease, and the plasma levels of PC (32:1 and 40:5) and PE (38:3, 38:4, 38:6, 40:4, 40:5, and 40:6) are collectively associated with hypertension [47].

Using lipoprotein model particles, we have demonstrated that SM in the surface monolayer of the particles increases the acyl chain order and reduces the head group hydration, which may be due to the saturated acyl chains and the intra- and intermolecular hydrogen bonding between the amino and hydroxyl groups of SM molecules [48]. We have shown that SM in the particle surface monolayer decreases the binding of apoE to the particles and the apoE-mediated uptake of the particles by HepG2 human hepatoblastoma cells [7]. Additionally, the incorporation of SM into the particle surface potentiates the inhibitory effects of apoC-II and apoC-III on apoE-mediated cellular uptake of the particles [7]. Arimoto et al. have shown that SM in the particle surface monolayer reduces LPL-mediated lipolysis and delays the plasma clearance of the particles in rats [49]. These findings suggest that SM suppresses the removal of TG-rich lipoproteins from the circulation.

In atherosclerotic lesions, many types of cells secrete sphingomyelinase (SMase), which hydrolyzes SM to form Cer and induces the aggregation of LDL particles [50]. Aggregated LDL particles in human atherosclerotic lesions contain higher amounts of Cer than LDL particles in the plasma [51]. LDL particles aggregated by SMase have the potential to form macrophage foam cells [52]. We have also demonstrated that, without apolipoproteins, the formation of Cer in lipoprotein model particles by SMase markedly stimulates the particle uptake by J774 mouse macrophages, which is mediated by heparan sulfate proteoglycans and LDL receptor-related protein [53]. The cellular uptake of Cer-containing particles is further enhanced by apoE [53]. Moreover, we have found that Cer molecules form globular, but not in-plane, microdomains in the particles and increase the binding of apoE to the particles [54]. Based on these observations, Cer derived from SM in the lipoprotein particles is a key factor in atherogenesis.

### Phospholipid class quantifications

In recent years, high-performance liquid chromatography with electrospray ionization-tandem mass spectrometry has been widely used for the identification and quantification of PL molecular species with different acyl chain compositions. However, because the ionization efficiencies in mass spectrometry analysis are different among PL molecular species, precise quantification of each PL molecular species requires many calibration curves [55]. The ionization efficiency markedly decreases with increasing acyl chain length but increases with increasing degree of acyl chain unsaturation [56, 57]. This problem has not been resolved yet. Therefore, the development of simple, high-throughput methods for quantifying PL classes is highly desired.

**Fig. 8:**
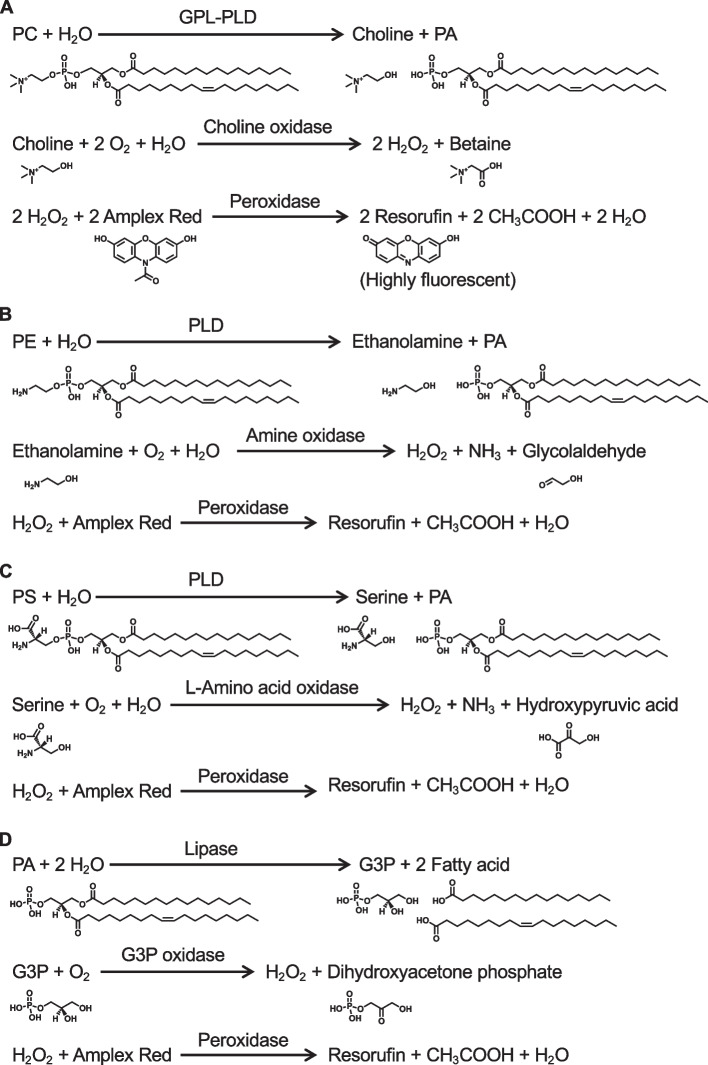
Reaction steps for enzymatic fluorometric assays of PC (**A**), PE (**B**), PS (**C**), and PA (**D**). In these assays, the final product, resorufin, is highly fluorescent and measurable. GPL-PLD, glycerophospholipid-specific phospholipase D; G3P, glycerol-3-phosphate; PA, phosphatidic acid; PC, phosphatidylcholine; PE, phosphatidylethanolamine; PLD, phospholipase D; PS, phosphatidylserine

**Fig. 9:**
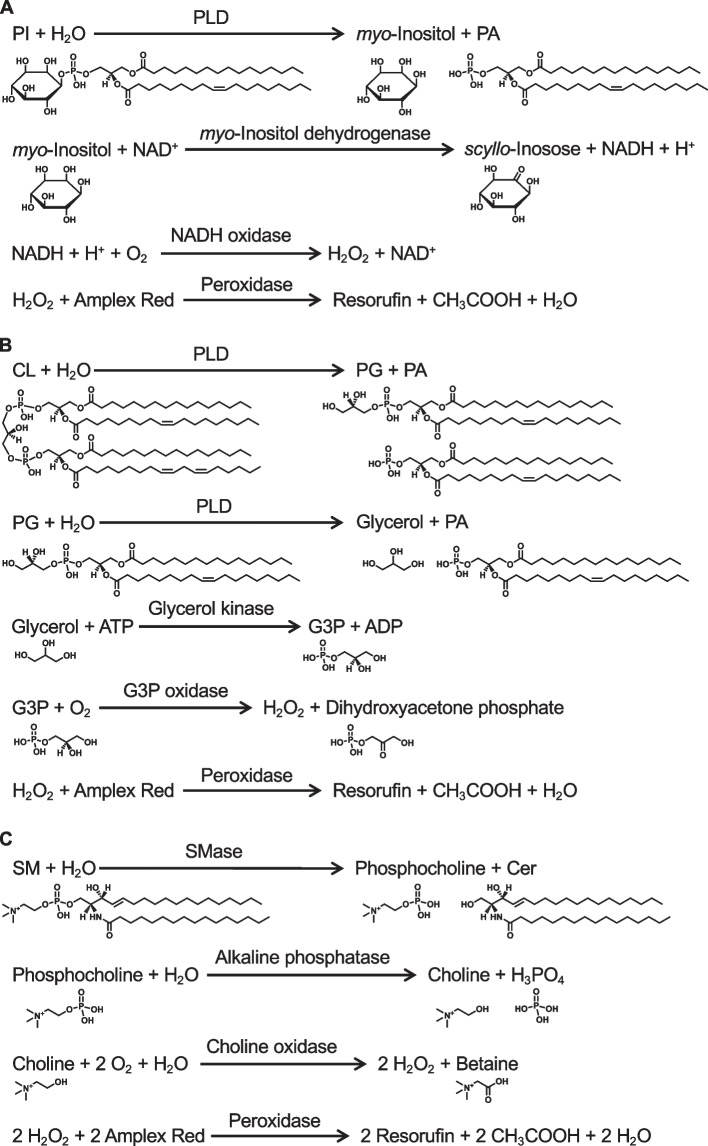
Reaction steps for enzymatic fluorometric assays of PI (**A**), PG + CL (**B**), and SM (**C**). In these assays, the final product, resorufin, is highly fluorescent and measurable. Cer, ceramide; CL, cardiolipin; G3P, glycerol-3-phosphate; PA, phosphatidic acid; PG, phosphatidylglycerol; PI, phosphatidylinositol; PLD, phospholipase D; SM, sphingomyelin; SMase, sphingomyelinase

To further clarify the physiological and pathological functions of PLs, we have developed enzymatic fluorometric assays to quantify all major PL classes, including PC, PE, PS, PA, PI, PG + CL, and SM [58–64]. Figures 8 and 9 show the principles of the enzymatic fluorometric assays using several specific enzymes and 10-acetyl-3,7-dihydroxyphenoxazine (Amplex Red) as a fluorogenic probe, which involve 3–5 steps. In the final steps of the assays, enzymatically produced hydrogen peroxide is detected using peroxidase and Amplex Red. In the presence of peroxidase, hydrogen peroxide and Amplex Red are converted to highly fluorescent resorufin (excitation maximum at 571 nm and emission maximum at 585 nm), acetic acid, and water [65]. Table 1 briefly summarizes the protocols for the enzymatic fluorometric assays for PL class quantification, which enable simple and high-throughput measurements by using a fluorescence microplate reader. These assays are accurate and sensitive with detection limits of 5–50 pmol (Table 1). The assay specificities depend on the enzymes used; however, the measurement of each PL class is not affected by the acyl chain length or double bond numbers. The enzymatic fluorometric assay for PC detects PC and plasmanylcholine, but not LPC or SM [59]. The enzymatic assay for PE detects PE, LPE and plasmenylethanolamine, but not PC or PS [59]. The enzymatic assay for PS detects PS and LPS, but not PC or PE [60]. The PA enzymatic assay does not distinguish between PA and LPA [58]. We have also developed the enzymatic fluorometric assays for LPA measurement, and thus, the concentration of PA can be determined by subtracting the concentration of LPA from the total concentration of PA and LPA [58]. The enzymatic assay for PI detects PI, LPI, PI(4)P, and PI(5)P, but not PI(3)P, PI(3,4)P_2_, PI(3,5)P_2_, PI(4,5)P_2_, or PI(3,4,5)P_3_ [63]. The PG + CL assay measures the sum of PG and CL concentrations and also detects LPG [62]. The enzymatic assay for SM detects only SM, but not sphingosylphosphocholine, PC, or LPC [61]. The principles and detailed protocols of enzymatic fluorometric assays have been described in our previous reports and review [58–64].

**Table 1:** Protocols for enzymatic fluorometric assays for quantification of major phospholipid classes

Assay	Reagent	Enzyme	Incubation	Heating	Detection limit
PC	C1	GPL-PLD	37 °C, 30 min		10 pmol
	C2	Choline oxidase Peroxidase	RT, 30 min		
PE	E1	PLD	37 °C, 30 min		10 pmol
	E2	Amine oxidase Peroxidase	RT, 30 min		
PS	S1	PLD L-Amino acid oxidase	25 °C, 240 min		50 pmol
	S2	Peroxidase	RT, 15 min		
PA	A1	Lipase	37 °C, 60 min	96 °C, 3 min	50 pmol
	A2	G3P oxidase Peroxidase	RT, 30 min		
PI	I1	PLD	37 °C, 60 min	96 °C, 3 min	20 pmol
	I2	*myo*-Inositol dehydrogenase	25 °C, 120 min		
	I3	NADH oxidase Peroxidase	45 °C, 60 min		
PG + CL	L1	PLD	37 °C, 30 min		10 pmol
	L2	Glycerol kinase G3P oxidase Peroxidase	RT, 30 min		
SM	M1	SMase Alkaline phosphatase	37 °C, 30 min		5 pmol
	M2	Choline oxidase Peroxidase	RT, 30 min		

*CL* cardiolipin, *GPL-PLD* glycerophospholipid-specific phospholipase D, *G3P* glycerol-3-phosphate, *PA* phosphatidic acid, *PC* phosphatidylcholine, *PE* phosphatidylethanolamine, *PG* phosphatidylglycerol, *PI* phosphatidylinositol, *PLD* phospholipase D, *PS* phosphatidylserine, *RT* room temperature, *SM* sphingomyelin, *SMase* sphingomyelinase

**Fig. 10:**
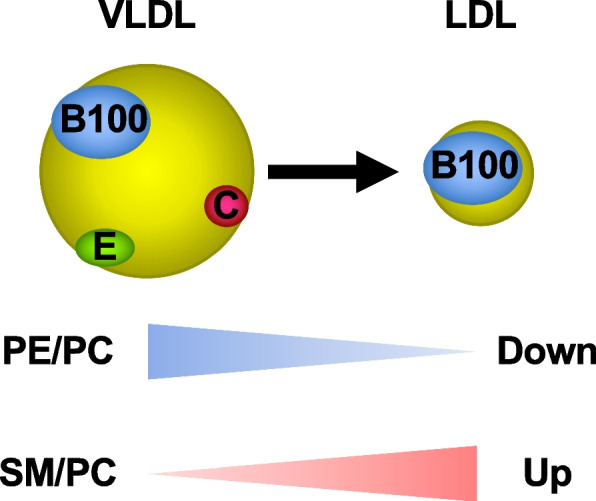
Phospholipid compositions of VLDL and LDL. During the conversion from VLDL to LDL, the PE/PC ratio decreases, but the SM/PC ratio increases. LDL, low density lipoprotein; PC, phosphatidylcholine; PE, phosphatidylethanolamine; PL, phospholipid; SM, sphingomyelin; VLDL, very low density lipoprotein

We have applied these enzymatic fluorometric assays for PL classes to various studies [66–74]. Furthermore, we have recently established and validated the enzymatic fluorometric methods for PC, PE, and SM measurements in human plasma VLDL, LDL, and HDL [32]. Using these enzymatic fluorometric assays, we have shown that the ratios of SM/PC are in the order VLDL (0.113) < HDL (0.164) < LDL (0.297), whereas the ratios of PE/PC are in the order VLDL (0.092) > HDL (0.051) > LDL (0.039) [32]. These results suggest the dilution of PE and condensation of SM in the particle surface monolayer during the conversion from VLDL to LDL (Fig. 10).

## Conclusions

The high level of LDL-cholesterol and low level of HDL-cholesterol are well-known risk factors for coronary heart disease. Lowering LDL-cholesterol by statins reduces the risk for coronary heart disease. On the other hand, the associations of plasma PL classes with coronary heart disease have been reported in several studies but have not yet been established. To facilitate the investigation of the roles of PL classes in various physiological processes, we have developed the assays for all major PL classes using combinations of specific enzymes and Amplex Red, which enable simple, accurate, sensitive, and high-throughput quantification. Comprehensive characterization of the PL compositions can be achieved using the combination of the enzymatic fluorometric assays and liquid chromatography-tandem mass spectrometry. Our developed enzymatic fluorometric assays will help to clarify the relationships of PL classes in plasma lipoproteins with coronary heart disease and to identify PL biomarkers.

## Data Availability

Not applicable.

## Abbreviations

apo: Apolipoprotein
CE: Cholesteryl ester
Cer: Ceramide
CETP: Cholesteryl ester transfer protein
CL: Cardiolipin
CM: Chylomicron
DG: Diglyceride
FC: Free cholesterol
GPL: Glycerophospholipid
HDL: High density lipoprotein
LCAT: Lecithin:cholesterol acyltransferase
LDL: Low density lipoprotein
Lp(a): Lipoprotein (a)
LPA: Lysophosphatidic acid
LPC: Lysophosphatidylcholine
LPE: Lysophosphatidylethanolamine
LPG: Lysophosphatidylglycerol
LPI: Lysophosphatidylinositol
LPS: Lysophosphatidylserine
LPL: Lipoprotein lipase
MTP: Microsomal triglyceride transfer protein
PA: Phosphatidic acid
PC: Phosphatidylcholine
PC-O: Ether-linked phosphatidylcholine
PCSK9: Proprotein convertase subtilisin-kexin type 9
PE: Phosphatidylethanolamine
PE-O: Ether-linked phosphatidylethanolamine
PG: Phosphatidylglycerol
PI: Phosphatidylinositol
PL: Phospholipid
PS: Phosphatidylserine
SM: Sphingomyelin
SMase: Sphingomyelinase
SPL: Sphingophospholipid
TG: Triglyceride
VLDL: Very low density lipoprotein
